# Vitamin B_12_ Status in Metformin Treated Patients: Systematic Review

**DOI:** 10.1371/journal.pone.0100379

**Published:** 2014-06-24

**Authors:** Qilin Liu, Sheyu Li, Heng Quan, Jianwei Li

**Affiliations:** Department of Endocrinology and Metabolism, West China Hospital of Sichuan University, Chengdu, China; University of Michigan Medical School, United States of America

## Abstract

**Objective:**

Randomized controlled trials and observational studies have yielded inconsistent results on the effects of metformin on vitamin B_12_ reduction. We therefore performed a systematic review to analyze the effects of metformin on vitamin B_12_ concentration.

**Methods:**

PubMed, Medline, Embase, and the Cochrane central registry of controlled trials were searched to identify randomized controlled trials and observational studies exploring the association between metformin and vitamin B_12_ concentration in patients with type 2 diabetes mellitus or polycystic ovary syndrome. The main outcome measure was changes in serum vitamin B_12_ concentration after 6–208 weeks of treatment with metformin, as compared with placebo or other anti-hyperglycemic therapy.

**Results:**

Six randomized controlled trials met the inclusion criteria. Serum vitamin B_12_ concentrations were significantly lower in patients treated with metformin than in those who received placebo or rosiglitazone (mean difference [MD], −53.93 pmol/L; 95% confidence interval [CI], −81.44 to −26.42 pmol/L, P = 0.0001). Subgroup analysis identified four trials in which patients received a lower dose of metformin (<2000 mg/d) and two in which they received a higher dose (≥2000 mg/d), with MDs in vitamin B_12_ concentration after metformin treatment of −37.99 pmol/L (95% CI, −57.44 to −18.54 pmol/L, P = 0.0001) and −78.62 pmol/L (95% CI, −106.37 to −50.86 pmol/L, P<0.00001), respectively.

**Conclusions:**

The reduction of vitamin B_12_ may be induced by metformin in a dose dependent manner.

## Introduction

Metformin is now the most widely used antidiabetic drug, with almost all guidelines throughout the world recommending metformin as first-line treatment for patients with type 2 diabetes mellitus (T2DM). Metformin may also be used to treat other conditions involving insulin resistance, such as polycystic ovary syndrome (PCOS) [1]. Metformin has beneficial effects on carbohydrate metabolism, weight loss, and vascular protection [2], but also has important side effects. For example, patients on long-term metformin therapy were found to be at risk of anemia [3]. This may be due to a metformin related vitamin B_12_ reduction. It is reported that, 30% of patients receiving long-term metformin treatment experienced malabsorption of vitamin B_12_, with a decrease in serum vitamin B_12_ concentration of 14% to 30% [4].

Vitamin B_12_ is a vital nutrient for health. It plays an important role in the functioning of the brain and nervous system, and in the formation of red blood cells. In addition to anemia, vitamin B_12_ deficiency may increase the severity of peripheral neuropathy in patients with T2DM [5]. Furthermore, because vitamin B_12_ participates in the most important pathway of homocysteine (Hcy) metabolism, a reduction in vitamin B_12_ would increase plasma concentrations of Hcy, which is strongly linked to cardiovascular disease in patients with T2DM [6] and PCOS [7].

Although some clinical studies have reported that metformin lowered vitamin B_12_ level, other studies have reported that it did not. To date, no consensus has been reached on whether metformin induces vitamin B_12_ reduction. We therefore performed a meta-analysis to assess the association between metformin treatment and vitamin B_12_ reduction.

## Methods

This systematic review was planned, conducted, and reported in accordance with the Preferred Reporting Items for Systematic Reviews and Meta-Analyses (PRISMA) Statement (**Table S1**) and the Cochrane handbook for Systematic Reviews of Interventions [8], [9].

### Inclusion criteria

We included studies that met the following criteria: (1) It should include patients with T2DM or PCOS who met strict diagnostic criteria and did not take B group vitamins prior to entrance into the studies. (2) For randomized controlled trials (RCTs), patients should be randomized to treatment with metformin or to placebo or another hypoglycemic drug and the groups compared by valid statistical methods. For observational studies, a group treated with metformin should be compared with another group treated with placebo or other antidiabetic drugs. (3) The main outcome should be change in serum vitamin B_12_ concentration. (4) All studies should report factors associated with changes in vitamin B_12_ levels necessary to perform a meta-analysis or sufficient information to estimate it.

### Exclusion criteria

Studies without available data, duplicate publications, and studies in a language other than English were excluded.

### Search strategy

The PubMed, Embase, and Cochrane central registry of controlled trials were systematically searched for all papers published through October 2013. Subject headings were combined with keywords and their synonyms, using search terms such as ‘metformin’, ‘Glucovance’, ‘dimethylbiguanid’, ‘vitamin B_12_’, ‘B_12_’, and ‘cobalamin’. References in selected articles and published reviews were also manually searched. Literature searches were performed independently by two investigators, with discrepancies resolved by group discussions. Additional studies and missing information in published reports were searched via direct author contact. Complete search strategy is reported as **Appendix S1**.

### Validity assessment

The validity of the eligible RCTs were evaluated in accordance with the Cochrane Collaboration guidance [8] which includes the following criteria: (1) random sequence generation, (2) allocation concealment, (3) blinding of participants and personnel, (4) blinding of outcome assessment, (5) incomplete outcome data, (7) selective reporting, and (8) other bias. For each criterion, an answer of ‘Yes’ indicated low risk of bias, ‘No’ indicated high risk of bias, and ‘Unclear’ indicated either lack of information or uncertainty over the potential for bias. We also applied the Newcastle-Ottawa Scale (NOS) [8], [10] to assess the quality of the included observational studies.

### Data extraction

Titles and abstracts were screened to identify clinical trials and observational studies. Full text articles of studies that fulfilled the inclusion criteria were obtained. Data obtained from each article included its title, author names, year of publication, study design, participant characteristics, and endpoint data. A meta-analysis was performed to examine the association between metformin treatment and the changes of vitamin B_12_ concentration. Vitamin B_12_ concentrations and changes were measured as pmol/L. Heterogeneity among the studies was evaluated using chi-square test and I^2^ statistics, with P≤0.1 and I^2^>50% indicating heterogeneity. A fixed effects model was selected for non-significant heterogeneity, or a random effects model for heterogeneity. The mean difference (MD) in each study was determined. The MDs were combined, and the pooled MDs along with their 95% confidence intervals (CIs) were calculated using Review Manager (RevMan, version 5.2). Subgroup analyses were performed by separating studies according to comparators, follow-up time, and background treatment. Tests for overall effect were assessed using z-statistics.

## Results

### Search results

**Figure 1:**
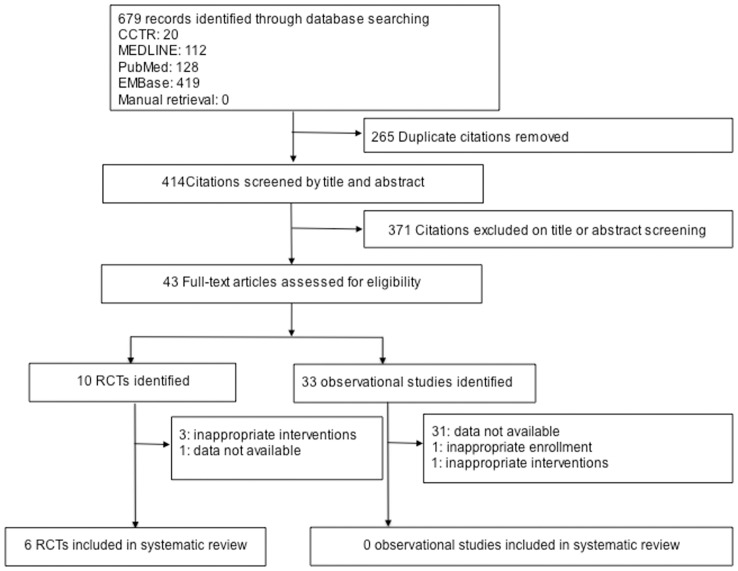
Flow-chart of the study.

A total of 679 articles were identified during our search. After screening of titles and abstracts, 43 met the criteria of our review, which were aimed at determining the influence of metformin on vitamin B_12_ status (**Figure 1**). Of these, 10 articles were RCTs and 33 were observational studies. Of the 10 RCTs, three reports were excluded because both groups received metformin as background treatment, and one was excluded because no major indices could be extracted, and eventually only six randomized controlled trials [11]–[16] met the inclusion criteria. Among the 33 observational studies, 15 were conference abstracts without available full texts [17]–[31] which could not provide sufficient information for quality assessment or data analysis, and 14 cross-sectional studies [3], [32]–[44] and 1 case-control study [5] lacked data of changes in serum vitamin B_12_ concentration from baseline, but vitamin B_12_ concentration changes had been defined as the primary outcome of this study. Thus, 30 studies were excluded. And the other 3 observational studies were cohort studies. One study compared metformin with phenformin [45], one study did not give vitamin B_12_ concentration changes [46], and the other one study enrolled the patients taken B group vitamins supplements [47]. Therefore, none of the observational studies met the inclusion criteria.

### Study characteristics

The characteristics of the six RCTs included in the final meta-analysis are shown in **Table S2**. All six were published between 2000 and 2010. Overall, they included 6 cohorts and 816 participants, with 610 completing the studies. The mean ages of the participants ranged from 24.1 to 64.0 years. The mean follow-up duration ranged from 6 to 208 weeks. Quality assessment of these studies by the Cochrane Collaboration Risk of Bias Tool is shown in **Figure S1**. All of the 6 RCTs were adequate randomized without selective reporting and other biases. Allocation concealment of 2 studies [11], [15], blinding of outcome assessment of 3 studies [13]–[15], and incomplete outcome data of 1 study [15] were all not described in details. In addition, blinding of participants and personnel was probably not been done in 2 studies [13], [15].

### Metformin and vitamin B_12_ reduction

**Figure 2:**
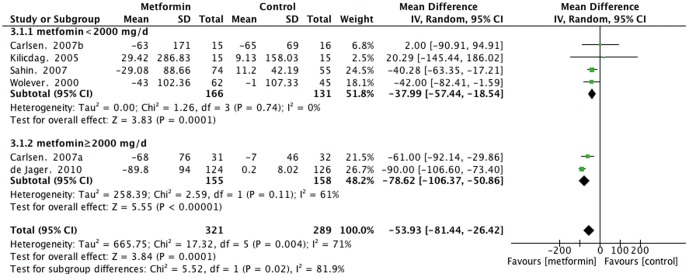
Effects of metformin on compared with other interventions vitamin B_12_ concentration.

Overall, metformin had a significant effect on vitamin B_12_ concentration when compared with other interventions (MD, −53.93 pmol/L; 95% CI, −81.44 to −26.42 pmol/L, P = 0.0001; **Figure 2**). However, we detected significant heterogeneity among the studies. The two trials [13], [14] with the lowest weight (total weight 9.3%) did not show a significant effect. The funnel plot was limited as only six trials were included.

As each 1000 mg/d metformin dose increment increased the risk of developing vitamin B_12_ deficiency [4], we chose metformin 2000 mg/d as the cut-off point in our subgroup analysis. The four trials [11], [13]–[15] belonging to the lower dose group showed that metformin had a significant effect compared with other interventions (MD, −37.99 pmol/L; 95%CI, −57.44 to −18.54 pmol/L, P = 0.0001), without any heterogeneity. The other two trials [14], [16] belonging to the higher dose group also showed a significant effect (MD, −78.62 pmol/L; 95%CI, −106.37 to −50.86 pmol/L, P<0.00001), with significant heterogeneity. We found that vitamin B_12_ concentration was reduced more in the higher than in the lower dose group, indicating that the reduction of vitamin B_12_ was associated with metformin dose.

We also assessed subgroups based on differences in duration of metformin use, background therapy, control group treatments, diseases of participants, countries, methods of B_12_ measuring and qualities of RCTs (**Table S3**). Subgroup analysis by diseases of participants showed that the effect of metformin on vitamin B_12_ concentration was nearly the same in patients with T2DM and PCOS. Moreover, subgroup analysis also indicated that metformin reduced vitamin B_12_ concentration in both long (≥3 years) and short (<3 years) treatment duration subgroup. All subgroups analysis according to any parameters, which may lead to heterogeneity, showed similar effects of metformin on vitamin B_12_ reduction.

### Adverse events

Two studies of RCTs didn't report any adverse event [11], [15]. Among the other 4 studies, gastrointestinal side effects were the most common adverse events [13], [14], [16]. In Carlsen's research of infertile PCOS women, the incidence of minor gastrointestinal side effects was 55.6% in the metformin group but 13.5% in the placebo group [14]. In his study of pregnant PCOS women, nausea and gastrointestinal discomfort was also found in 17.6% patients treated with metformin and 14.3% patients treated with placebo [14]. In Kilicdag's study in T2DM patients [13], none of the patients with rosiglitazone reported any adverse effects, but about 20% patients treated with metformin had problems of nausea and vomiting. De Jager et al. [16] found 46 of 390 T2DM participants (30 with metformin, 16 with placebo) experienced adverse events such as diarrhea, flatulence, fatigue, pruritus, headaches, heartburn and nausea. Additionally, 11.3% patients with metformin but 5.6% patients with placebo reported a history of diarrhea. However, there was no significant difference in the incidence of other side effects. In general, gastrointestinal side effects were more common in patients treated with metformin. Adverse events of the 6 RCTs were shown in **Table S4**.

## Discussion

Overall, we found that metformin treatment of patients with diabetes and PCOS resulted in statistically significant reductions in vitamin B_12_ concentration. Although none of the 33 observational studies met the inclusion criteria, almost all of these observational studies supported that metformin exposure was associated with a significant reduction of vitamin B_12_. For example, Kos' cohort study [47] found vitamin B_12_ levels of T2DM patients treated with metformin for more than 4 years were significantly lower than control (MD, −152.2 pg/mL; 95%CI, −220 to −84 pg/mL, P<0.0001). And Greibe's cohort study [46] demonstrated that, compared with placebo, serum vitamin B_12_ in women with Polycystic Ovary Syndrome treated with metformin was decreased after 6 months of treatment. Besides, cross-sectional studies and surveys [41], [44] also showed a significant effect of metformin therapy on vitamin B_12_ reduction, when compared with other hypoglycemic therapy.

In consistent with the observational studies, meta-analysis of RCTs also found that metformin reduced vitamin B_12_ levels in patients with diabetes and PCOS. Subgroup analysis showed higher-dose metformin could reduce vitamin B_12_ concentration more significantly. Our meta-analysis included RCTs of patients with PCOS in addition to T2DM, because both diseases have a common pathogenesis, i.e., insulin resistance. Subgroup analysis showed that metformin had nearly the same effects on vitamin B_12_ in patients with T2DM or PCOS.

Because metformin, which delays glucose absorption, has an effect on small bowel motility and on bacterial overgrowth [48]. According to our review, gastrointestinal adverse effects were the frequently observed in patients treated with metformin. Metformin-induced B_12_ malabsorption may be due to digestive changes, which leads to the binding of B_12_-intrinsic factor (IF) complex and a reduction of B_12_ absorption [49]. Since the B_12_-IF complex binds to the ileal cell surface receptor, metformin alterations in IF levels and/or ileal morphological structure may lead to B_12_ reduction [50], [51].

The clinical significance of biochemical change in serum vitamin B_12_ concentrations remains controversial. Some previous studies showed that lower serum B_12_ concentration caused by metformin within the normal range could be clinically meaningful. A Greek cohort including 600 diabetic patients showed association between metformin prescription and vitamin B_12_ dependent megaloblastic anemia [3]. Cognitive impairment and the progression of diabetic peripheral neuropathy may also be accelerated by metformin in a vitamin B_12_ dependent manner [52]. So, it was implied that the decrease of serum B_12_ concentration within the normal range should not be overlooked.

However, some studies showed that metformin may even improve B_12_ metabolism [43], or only decreases the inactive form of vitamin B_12_ (known as holo-haptocorrin, holoHC), rather than the biological active form (known as holo-haptocorrin, holoTC) [46], [53]. On the other hand, some other studies just provided the opposite results [5], [35] and the lowered serum level of vitamin B_12_ caused by metformin was reported to be clinically meaningful.

Different countries have different eating habits and nutrition supplements. And prevalence of vitamin B_12_ deficiency varies with population and B_12_ cut-off used. According to a national survey from U.S., consumption of routine vitamin B_12_ supplement may not rescue the biochemical B_12_ reduction in diabetic patients with metformin usage, suggesting that the recommended amount of vitamin B_12_ by the Institute of Medicine (IOM) may not be sufficient in those using metformin [41]. However, several studies [54], [55] found that treatment with vitamin B_12_ in a higher dose could reverse some of these disadvantages and vitamin B_12_ deficiency. Therefore, the supplement of vitamin B_12_ may be reasonable, and the dose should be individualized according to different ethnicity and habits.

Our study has several limitations. First, although we included patients with PCOS or T2DM, it is not clear whether these diseases cause vitamin B_12_ reductions independently. Second, the examining methods of vitamin B_12_ concentration were diverse in the included studies. Third, we only searched for articles published in English, while studies published in other languages could not be analyzed according to our knowledge. Moreover, potential publication bias could be introduced due to negative results may be rejected by journals. Finally, we did not analyze confounding factors that may introduce heterogeneity, such as obesity and age.

In conclusion, our meta-analysis showed that metformin could reduce vitamin B_12_ levels in a dose-dependent manner. Since vitamin B_12_ is essential to nutrition, metformin-induced B_12_ reduction may have detrimental effects in patients with T2DM and PCOS. Patients treated with metformin may benefit from vitamin B_12_ supplements. Because few clinical studies have assessed this directly, additional, well-designed trials are needed to confirm our findings.

## Supporting Information

Figure S1 **Quality assessment of RCTs.**
(TIF)

Table S1 **PRISMA checklist.**
(DOC)

Table S2 **Study characteristics.**
(DOC)

Table S3 **Subgroup analysis.**
(DOC)

Table S4 **Adverse events.**
(DOC)

Appendix S1 **Search strategy.**
(DOC)
