# Supplementary figures and images for: Vitamin B12 Status in Metformin Treated Patients: Systematic Review
Source: PLoS One. 2014 Jun 24;9(6):e100379. doi: 10.1371/journal.pone.0100379 (PMC4069007; doi:10.1371/journal.pone.0100379)

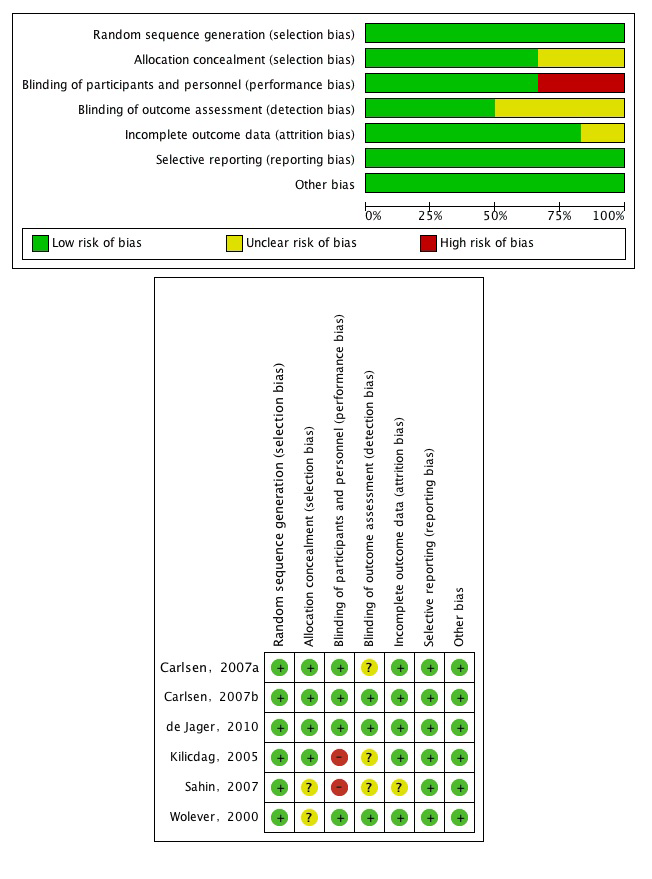

Supplement: Figure S1 — Quality assessment of RCTs. (TIF) [file pone.0100379.s001.tif]
