# Supplementary material for: Vitamin B12 Status in Metformin Treated Patients: Systematic Review
Source: PLoS One. 2014 Jun 24;9(6):e100379. doi: 10.1371/journal.pone.0100379 (PMC4069007; doi:10.1371/journal.pone.0100379)
Supplement: Table S2 — Study characteristics. (DOC) [file pone.0100379.s003.doc]

| Study characteristics | Wolever, 2000 [11] | Kilicdag, 2005 [13] | Carlsen, 2007 a [14]* | Carlsen, 2007 b [14]* | Sahin, 2007 [15] | Wulffele, 2003[12]**  De Jager, 2010[16]** |
| --- | --- | --- | --- | --- | --- | --- |
| Country | Canada | Turkey | Norway | Norway | Turkey | Netherlands |
| Patients,N  (I/C) | 83/83 | 15/15 | 31/32 | 15/16 | 74/55 | 196/194 |
| BMI, kg/m^2^  (I/C) | 30.5/30.8 | 26.2/29.3 | 28.6/29.9 | 32.1/29.3 | 29.1/29.6 | 30/30 |
| Age,Year  (I/C) | 58.7/58.5 | 24.1/25.5 | 28.9/30.2 | 28.9/28.3 | 58.4/57.9 | 64/59 |
| Participants | T2DM | PCOS | PCOS  (Infertile) | PCOS  (Pregnant) | T2DM | T2DM |
| Women,%  (I/C) | 20.5/21.7 | All women | All women | All women | 58.1/63.6 | 57.7/49.4 |
| Intervention | Metformin  (1500 mg/day) | Metformin  (1700 mg/day) | Metformin  (2000 mg/day) | Metformin  (1700 mg/day) | Metformin  (1700 mg/day) | Metformin***  (mean, 2163 mg/day) |
| Control | Placebo | Rosiglitazone  (4 mg/day) | Placebo | Placebo | Rosiglitazone  (4 mg/day) | Placebo |
| Primary treatment | Oral glucose lowering drugs | N | N | N | Lifestyle intervention | Insulin/oral glucose lowering drugs |
| Washout period | 8 weeks placebo | N | N | N | 4 weeks | 12 weeks insulin |
| Background treatment | Lifestyle intervention | Lifestyle intervention | Folic acid  (0.4 mg/day); One multivitamin tablet/day | Folic acid  (1.0 mg/day);  One multivitamin tablet/day | Lifestyle intervention | Insulin |
| Follow-up | 36 weeks | 12 weeks | 16 weeks | 36 weeks | 6 weeks | 208 weeks |
| Measures | A | B | C | C | B | B |

**Table S2. Study characteristics of RCTs**

*This article included two separate RCTs.

**The two studies were short-term and long-term outcomes of the same trial. The long-term outcomes (De Jager, 2010) will be included into our research.

***Each patient in this group maintained his/her maximally tolerated daily dose (one, two or three tablets of 850 mg) during the trial; the actual mean dose in the metformin-treated group was 2163 mg/day.

A: Radioimmunoassay; B: Electrochemiluminiscence immunoassay; C: Competitive protein binding assay.

I: intervention group; C: control group.
