# Supplementary material for: Vitamin B12 Status in Metformin Treated Patients: Systematic Review
Source: PLoS One. 2014 Jun 24;9(6):e100379. doi: 10.1371/journal.pone.0100379 (PMC4069007; doi:10.1371/journal.pone.0100379)
Supplement: Table S3 — Subgroup analysis. (DOC) [file pone.0100379.s004.doc]

- **Table S3. Subgroup analysis**

| - Subgroups | Study  N | | - MD（95%CI） - pmol/L | Heterogeneity test | | - Model | Overall effect test   - P |
| --- | --- | --- | --- | --- | --- | --- | --- |
|  |  |  |  | P | I^2^ |  |  |
| - Duration of metformin use | | | | | | | |
| - <3 years | | - 5 | - −55.87 (-87.75, -23.99) | - 0.003 | - 75% | - R | - 0.0006 |
| - ≥3 years | | - 1 | - −42.00 (-82.41, -1.59) | - --- | - --- | - --- | - 0.04 |
| - Background therapy | | | | | | | |
| - Folic acid | | - 2 | - −54.64 (-84.16, -25.11) | - 0.21 | - 37% | - F | - 0.0003 |
| - Insulin | | - 1 | - −90.00 (-106.60, -73.40) | - --- | - --- | - --- | - <0.00001 |
| - Lifestyle interventions | | - 3 | - −39.82 (-59.72, -19.93) | - 0.77 | - 0% | - F | - <0.0001 |
| - Controls | | | | | | | |
| - Placebo | | - 4 | - −61.75 (-92.54, -30.96) | - 0.03 | - 67% | - R | - <0.0001 |
| - Rosiglitazone | | - 2 | - −39.13 (-61.98, -16.28) | - 0.48 | - 0% | - R | - 0.0008 |
| - Diseases | | | | | | | |
| - Type 2 diabetes mellitus | | - 3 | - −59.34 (-97.12, -21.57) | - 0.001 | - 85% | - R | - 0.002 |
| - Polycystic ovary syndrome | | - 3 | - −45.17 (-86.69, -3.64) | - 0.31 | - 15% | - R | - 0.03 |
| - Countries | | | | | | | |
| - Norway | | - 2 | - −54.64 (-84.16, -25.11) | - 0.21 | - 37% | - R | - 0.0003 |
| - Turkey | | - 2 | - −39.13 (-61.98, -16.28) | - 0.48 | - 0% | - R | - 0.0008 |
| - Canada | | - 1 | - −42.00 (-82.41, -1.59) | - --- | - --- | - --- | - 0.04 |
| - Netherlands | | - 1 | - −90.00 (-106.60, -73.40) | - --- | - --- | - --- | - <0.00001 |
| - Measurements | | | | | | | |
| - Dual count solid phase no boil assay | | - 1 | - −42.00 (-82.41, -1.59) | - --- | - --- | - --- | - 0.04 |
| - Competitive protein binding assay | | - 2 | - −45.33 (-98.71, -8.05) | - 0.21 | - 37% | - R | - 0.10 |
| - Electrochemiluminiscence immunoassay | | - 3 | - −59.85 (-106.78, -12.93) | - 0.002 | - 85% | - R | - 0.01 |
| - Quality of RCTs | | | | | | | |
| - < 4 | | - 1 | - −40.28 (-63.35, -17.21) | - --- | - --- | - --- | - 0.0006 |
| - ≥ 4 | | - 5 | - −59.00 (-89.64, -28.37) | - 0.04 | - 61% | - R | - 0.0002 |
