# Supplementary material for: Vitamin B12 Status in Metformin Treated Patients: Systematic Review
Source: PLoS One. 2014 Jun 24;9(6):e100379. doi: 10.1371/journal.pone.0100379 (PMC4069007; doi:10.1371/journal.pone.0100379)
Supplement: Table S4 — Adverse events. (DOC) [file pone.0100379.s005.doc]

- **Table S4. Adverse events.**

| Study ID, year | Adverse events | Metformin group  N | Control group  N |
| --- | --- | --- | --- |
| Wolever, 2000 [11] | NR | NR | NR |
| Kilicdag, 2005 [13] | Nausea and vomiting | 3 | 0 |
| Carlsen, 2007a [14] | Minor gastrointestinal side effects | 20 | 5 |
| Carlsen, 2007b [14] | Nausea and gastrointestinal discomfort | 3 | 3 |
| Sahin, 2007 [15] | NR | NR | NR |
| De Jager, 2010 [16] | Diarrhea | 22 | 11 |
|  | Flatulence | 10 | 10 |
|  | Fatigue | 7 | 8 |
|  | Pruritus | 5 | 9 |
|  | Headaches | 6 | 9 |
|  | Heartburn | 7 | 9 |
|  | Nausea | 10 | 10 |

- NR: not reported; N: number.
