# Supplementary material for: Vitamin B12 Status in Metformin Treated Patients: Systematic Review
Source: PLoS One. 2014 Jun 24;9(6):e100379. doi: 10.1371/journal.pone.0100379 (PMC4069007; doi:10.1371/journal.pone.0100379)
Supplement: Appendix S1 — Search strategy. (DOC) [file pone.0100379.s006.doc]

Medline, Embase and the Cochrane central registry of controlled trials

1.exp metformin/

2.( riomet or siofor or dimethyl biguanid$ or dimethylbiguanid$ or dimethyldiguanide

or dimethylguanylguanid$ or aron or dabex or deson or dextin or diabex or diaformin$ or diamin or diformin$ or dimefor or dimefor or flu?amine or fortamet or glafornil or glifage or gluformin or glume$ or glupa or glyformin or glymet or haurymellin or i-max or la 6023 or meglucon or meguan or melbin or melformin or mescorit or metaformin or metiguanide or metphormin or neoform or diabetase or diabetmin or diabetosan or glucofage or glucoformin or glucohexal or glucoless or glucomet or glucomin or glyciphage or glycomet or glycon or glycon or glyformin or glymet or metfogamma or metforal or methformin or glucovance or metformin* or glucophag*).tw.

3.1 or 2

4. exp cyanocobalamin/

5. b12 or b 12 or antipernicin or bedoc or behepan or benol or berubigen or betolvex

or bevidox or bex or bexii or cobalamide or cobalin or cobaltron or cobamin or cobamine or cobione or cohemin or b-12 or cyanacobalamin or cyancobalamin or cyanocobalomin or cycobemin or cyomin or cytacone or cytagon or cytamen or cytobion or depinar or dicopac or dobetin or docemine or docigram or dodecavite or dodex or ducobee or ducobee or endoglobin or examen or extrinsic factor or fermin or fresmin or hepagon or hepavis or hepavit or macrabin or millevit or kaybovite or nascobal or pernical or poyamine or redisol or regividerm or rubion or rubivitan or rubramin or rubranova or rubrine or ruvite or sytobex or twelbe or twelve oral or vitarubin or virubra or cyano cobalamin or cn cobalamin or b complex or cobalamin$ or hydroxycobalamin$ or cyanocobalamin$).tw.

6. 4 or 5

7. 3 and 6

| pubmed |
| --- |
|  |
| #7,Search (#6) AND #3 |
| #6,Search (#5) OR #4 |
| #5,Search (((((((((((((((((((((((((((glucophag*[Text Word]) OR metformin*[Text  Word]) OR glucovance[Text Word]) OR glymet[Text Word]) OR glyformin[Text Word]) OR glycon[Text Word]) OR glycomet[Text Word]) OR glyciphage[Text Word]) OR diabetosan[Text Word]) OR diabetmin[Text Word]) OR metaformin[Text Word]) OR melformin[Text Word]) OR melbin[Text Word]) OR meguan[Text Word]) OR i-max[Text Word]) OR la 6023[Text Word]) OR glume*[Text Word]) OR diformin*[Text Word]) OR diamin[Text Word]) OR diaformin*[Text Word]) OR diabex[Text Word]) OR dimethylbiguanid*[Text Word]) OR dimethyldiguanide[Text Word]) OR dimethylguanylguanid*[Text Word]) OR aron[Text Word]) OR dimethyl biguanid*[Text Word]) OR siofor[Text Word]) OR neoform[Text Word] |
| #4,"Search ""Metformin""[Mesh] OR ""Glucovance"" [Supplementary Concept]" |
| #3,Search (#2) OR #1 |
| #2,Search (((((((((((((((((((((((((((((((((((((((((((cyanocobalamin*[Text Word]) OR  hydroxycobalamin*[Text Word]) OR cobalamin*[Text Word]) OR b complex[Text Word]) OR cn cobalamin[Text Word]) OR cyano cobalamin[Text Word]) OR virubra[Text Word]) OR rubrine[Text Word]) OR rubramin[Text Word]) OR rubivitan[Text Word]) OR rubion[Text Word]) OR redisol[Text Word]) OR nascobal[Text Word]) OR kaybovite[Text Word]) OR hepavis[Text Word]) OR fresmin[Text Word]) OR fermin[Text Word]) OR extrinsic factor[Text Word]) OR examen[Text Word]) OR endoglobin[Text Word]) OR ducobee[Text Word]) OR docigram[Text Word]) OR dicopac[Text Word]) OR depinar[Text Word]) OR cytobion[Text Word]) OR cytacone[Text Word]) OR cycobemin[Text Word]) OR cyancobalamin[Text Word]) OR cyanacobalamin[Text Word]) OR b-12[Text Word]) OR cohemin[Text Word]) OR cobione[Text Word]) OR cobamine[Text Word]) OR cobaltron[Text Word]) OR cobalamide[Text Word]) OR bexii[Text Word]) OR bex[Text Word]) OR bevidox[Text Word]) OR betolvex[Text Word]) OR berubigen[Text Word]) OR behepan[Text Word]) OR b 12[Text Word]) OR b12[Text Word]) OR cyanocobalamin[Text Word] |
| #1,"Search (""Vitamin B 12""[Mesh]) OR ""Vitamin B 12 Deficiency""[Mesh]" |
